# P01-15 National focal point network for physical activity promotion - experiences from the european union

**DOI:** 10.1093/eurpub/ckac095.015

**Published:** 2022-08-29

**Authors:** Antonina Tcymbal, Peter Gelius, Karim Abu-Omar, Sven Messing, Stephen Whiting, Wickramasinghe Kremlin

**Affiliations:** 1 Department of Sport Science and Sport, Friedrich-Alexander-Universität Erlangen-Nürnberg, Erlangen, Germany; 2 Division of Noncommunicable Diseases and Promoting Health through the Life-course, World Health Organization Regional Office for Europe, Moscow, Russia; 3 European Office for the Prevention and Control of Noncommunicable Diseases, World Health Organization, Moscow, Russia

**Keywords:** Physical activity policy, physical activity promotion, international collaboration

## Abstract

**Background:**

An analysis of currently existing partnerships and cross-country collaboration for physical activity (PA) promotion is valuable for understanding how such partnerships operate, and how they impact national PA promotion efforts. This study aimed to outline the structure of the European Union's (EU) National Physical Activity Focal Point Network, to evaluate its outputs and benefits, and to describe its potential and challenges.

**Methods:**

We employed a mixed methods approach with three components: (1) document analysis of network meeting reports, (2) semi-structured interviews with key officials who were involved in establishing the network, and (3) an online evaluation survey with the national PA Focal Points.

**Results:**

The PA Focal Point Network was founded in 2014, and its main task is to coordinate the collection of information for the EU's HEPA Monitoring Framework. Each of the EU Member States nominated a representative to the network. Focal Points usually meet twice a year to discuss issues related to the HEPA Monitoring Framework and to share best practices and plan activities for the promotion of PA within the EU. The results of the evaluation survey show that participation in the network helped members to specify goals for PA promotion, gain knowledge, and identify opportunities to promote PA in their country. From the perspective of the Focal Points, most helpful outputs of the Network activity are the country factsheets on physical activity, the connections within the Network and the opportunity to share their experience with colleagues during meetings and group discussions.

**Conclusions:**

The study shows that the PA Focal Point Network may serve as an example of successful cross-country collaboration in PA promotion. The network has supported the monitoring of the implementation of the EU Council Recommendation on HEPA across sectors in particular and of PA promotion in the EU in general. It also had positive effects on national PA promotion efforts and on cooperation between countries. All in all, the PA Focal Point Network can serve as an example for other world regions or policy areas that set up similar networks.

